# Copper (II) Acylhydrazinates. Their Synthesis and Characterization

**DOI:** 10.1155/MBD.2001.171

**Published:** 2001

**Authors:** Zahid H. Chohan, M. A. Farooq, Claudiu T. Supuran

**Affiliations:** 1 Department of Chemistry Islamia University Bahawalpur Pakistan; 2 Laboratorio di Chimica Inorgania e Bioinorganica Uriversita degli Studi Via Gino Capponi 7 Firenze 1-50121 Italy

## Abstract

Acylhydrazine derived furanyl and thienyl Schiff bases and their Cu(II) complexes have been prepared and characterized on the basis of their physical, spectral and analytical data. The preferred enolic form of the Schiff base function as a tetradentate ligand during coordination to the metal ion yielding a square planar complex. The Schiff bases and their complexes with different anions were tested for their antibacterial activity against bacterial species such as *Escherichia coli, Staphylococcus aureus, Pseudomonas aeruginosa andKlebsiella pneumonae*.


### COPPER(II) ACYLHYDRAZINATES. THEIR SYNTHESIS AND CHARACTERIZATION

Zahid H. Chohan*[1], M. A. Farooq[1] and Claudiu T. Supuran[2]

[1]Department of Chemistry, Islamia University, Bahawalpur, Pakistan
[2]Laboratorio di Chimica Inorganica e Bioinorganica, Universita degli Studi, Via Gino Capponi 7, I-50121, Firenze, Italy

### ABSTRACT
Acylhydrazine derived furanyl and thienyl Schiff bases and their Cu(II) complexes have been prepared and characterized on the basis of their physical, spectral and analytical data. The preferred enolic form of the Schiff base function as a tetradentate ligand during coordination to the metal ion yielding a square planar complex. The Schiff bases and their complexes with different anions were tested for their antibacterial activity against bacterial species such as *Escherichia coli*, *Staphylococcus aureus*, *Pseudomonas aeruginosa and Klebsiella pneumonae.*.

### INTRODUCTION
Many studies[1-4] have indicated the interesting and varied ligational behavior of hydrazine and hydrazones towards transition metal ions. Their bacteriostatic properties[5-9] were well studied by many researchers[10-12]. Some hydrazones have also been found to act as potent inhibitors for DNA synthesis in a variety of cultured human cells and in particular, their Cu(II) complexes were shown to produce significant inhibition of tumor growth when administered to mice bearing a transplanted fibrosarcoma[13,14]. Because of such promising results, the antibacterial metallo-organic chemistry of such ligands is yet to be explored. We have previously reported[15-18] some antibacterial acylhydrazine derived Schiff bases and their various transition metal complexes. The present study was undertaken in order to prepare and study the metallo-organic/coordination behavior of Cu(II) with the Schiff base ligands $L^1$ and $L^2$.

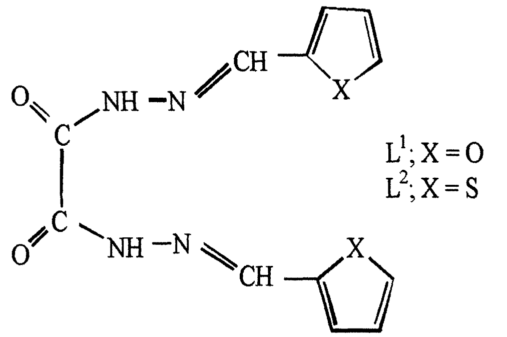

Structure of the Ligand

### EXPERIMENTAL
### Material and Methods
All chemicals and solvents used were of Analar grade. Cu(II) salts were used as nitrate, sulphate, oxalate and acetate. 2-Furanecarboxaldehyde, 2-thiophenecarboxaldehyde, oxaloyldihydrazide were obtained from E. Merck. UV-Visible spectra were obtained on a Hitachi U-2000 double-beam spectrophotometer, IR, on a Philips Analytical PU 9800 FTIR instrument. $^1H$ NMR and $^{13}C$ NMR spectra were recorded on a Brucker 250 MHz spectrometer. C, H and N analyses were carried out by Butterworth Laboratories Ltd. Conductances of the metal complexes were determined in DMF on a YSI-32 model conductometer. Magnetic measurements were done on solid complexes using the Gouy method. Melting points were recorded on a Gallenkamp apparatus without correction.

### Preparation of Schiff base Ligands
**N,N'-Bis(2-furanylmethylidene)oxaloyldihydrazide ($L^1$).**
2-Furanecarboxaldehyde (1.9 mL, 2.2 g, 0.02 mol) in absolute ethanol (20 mL) was added to a stirred hot ethanol solution (30 mL) of oxaloyldihydrazide (1.2 g, 0.01 mol). Then 2-3 drops of conc. $H_2SO_4$ were added and the mixture was refluxed for 8 h. The reaction mixture was then cooled and left for 24 h at room temperature. During this period, a light yellow solid was formed which was recrystallized from hot ethanol to give the desired product ($L^1$) (1.8 g). $L^2$ was prepared according to the same reported[17] method described as for $L^1$.

**Preparation of the Metal Complexes**

An ethanol solution (20 mL) of the appropriate Cu(II) salt (0.001 mol) was added to a well-stirred hot ethanol solution (20 mL) of the respective Schiff base (0.001 mol). The mixture was refluxed for 8 h. Then on cooling at room temperature; a precipitated solid product was formed. The product thus obtained was filtered, washed with ethanol, then with ether and dried. Crystallization from aqueous ethanol (50 %) gave the desired metal complexes.

### RESULTS AND DISCUSSION

**Physical Properties**

The Schiff bases ($L^1$ and $L^2$) (Fig. 1) were prepared by reacting the appropriate amount of 2-furanecarboxaldehyde and 2-thiophenecarboxaldehyde in hot ethanol with oxaloyldihydrazide in 1 : 2 molar ratio. The structures of these Schiff base ligands were established[17] with the help of IR, $^1$H NMR, $^{13}$C NMR spectroscopic and microanalytical data. All the metal complexes (Table I) were prepared by the stoichiometric reaction of Cu(II) salt and Schiff base ligands in equimolar ratio (M:L=1:1). The complexes are intensely colored and stable solids, which decompose above 200°C without melting and are insoluble in common organic solvents such as ethanol, methanol, chloroform or acetone. DMSO and DMF, however, dissolved all the complexes. Molar conductance values (92-98 $ohm^{-1}cm^2mol^{-1}$) of the soluble complexes in DMF show values indicating[19] that that they are all electrolytes.

Table I. Physical and Analytical Data of the Cu(II) Complexes

| Complex | M.p (°C) | B.M ($\mu_{eff}$) | Calc (Found) % C H N |
|---|---|---|---|
| 1 $[Cu(L^1)_2](NO_3)_2$ $C_{12}H_{10}CuN_6O_{10}$ [461.5] | 218-220 | 1.55 | 31.2 2.2 18.2 (31.6) (2.1) (18.5) |
| 2 $[Cu(L^1)_2](SO_4)$ $C_{12}H_{10}CuN_4O_8S$ [433.6] | 222-224 | 1.62 | 33.2 2.3 12.9 (33.4) (2.2) (13.0) |
| 3 $[Cu(L^1)_2](C_2O_4)$ $C_{14}H_{10}CuN_4O_8$ [425.5] | 220-222 | 1.57 | 39.5 2.4 13.2 (39.3) (2.6) (13.1) |
| 4 $[Cu(L^1)_2](CH_3CO_2)_2$ $C_{16}H_{14}CuN_4O_8$ [453.5] | 224-226 | 1.65 | 42.3 3.1 12.3 (42.5) (3.5) (12.2) |
| 5 $[Cu(L^2)_2](NO_3)_2$ $C_{12}H_{10}CuN_6O_8S_2$ [493.7] | 222-224 | 1.58 | 29.2 2.0 17.0 (29.1) (2.2) (17.4) |
| 6 $[Cu(L^2)_2](SO_4)$ $C_{12}H_{10}CuN_4O_6S_2$ [433.6] | 227-230 | 1.55 | 33.2 2.3 12.9 (33.6) (2.2) (12.6) |
| 7 $[Cu(L^2)_2](C_2O_4)$ $C_{14}H_{10}CuN_4O_6S_2$ [457.7] | 224-226 | 1.60 | 36.7 2.2 12.2 (36.9) (2.6) (12.1) |
| 8 $[Cu(L^2)_2](CH_3CO_2)_2$ $C_{16}H_{14}CuN_4O_6S_2$ [485.66] | 226-228 | 1.62 | 39.5 2.9 11.5 (39.3) (2.7) (11.3) |

Model studies of these Schiff bases (Fig. 2) show that in no case can these Schiff bases exhibit tridentate behavior. They are only capable of exhibiting tetradentate (Fig. 2) behavior. However, they have a tendency to exhibit different tautomers, either as diketone [Fig. 2A], dienol [Fig. 2B] and as well as ketoenol [Fig. 2C]. In the dienol form, the ligands may coordinate metal ions through X (X=O or S) donor sites of furanyl or thienyl and through the two azomethine nitrogens (HC=N). The diketone and ketoenol forms can also behave similarly, as tetradentate ligands, coordinating through the same coordination sites as dienol form.

**Infrared Spectra**

The IR spectra of the Schiff bases and their Cu(II) complexes were recorded in KBr and are reported in Tables I with some tentative assignments of their important characteristic bands. All these Schiff bases showed the absence of the bands at ~3420 $cm^{-1}$ and 1730 $cm^{-1}$ due to characteristic $\nu(NH_2)$ and $\nu(C=O)$ stretching vibrations of the respective hydrazinoamine and aldehyde. Instead, a new band at ~1635 $cm^{-1}$ assigned[20] to the azomethine (HC=N) linkage appeared in the spectra of all of the proposed Schiff base ligands. Also a band at 1020 $cm^{-1}$ due to a HN-N vibration appeared in the spectra of $L^1$ and $L^2$. This observation suggested[21] that the hydrazinoamine and aldehyde moieties of the starting reagents are no longer present and that condensation to the respective Schiff bases has taken place.

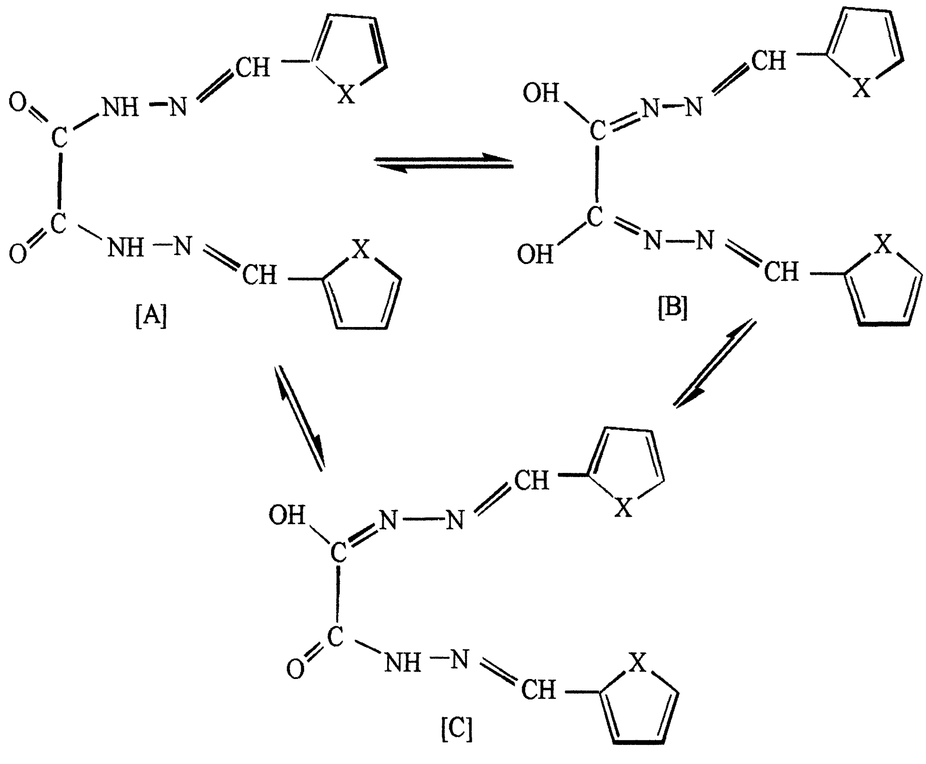

Fig. 2 Tautomeric forms of the investigated ligands

A comparison of the infrared spectra of the Schiff bases and their metal complexes indicated[21,22] that the Schiff bases are tetradentately coordinated to the metal ions. The band due to ν(C=O) was absent in the spectra of the complexes suggesting[23] enolization of the Schiff bases during complexation. This is supported by the evidence that the band due to ν(OH) in the spectra of these complexes was observed at ~3315 $cm^{-1}$. These facts suggested that the Schiff bases $L^1$ and $L^2$ remained in the keto form in the solid state as uncomplexed ligands but in solution the keto and enol forms were in equilibrium[24], as shown in Fig. 2B. The amide-II band was split, displaced to higher frequency and reduced in intensity. Shift (5-10 $cm^{-1}$) to higher frequency of the ν(N-N) band at 1025 $cm^{-1}$ and its splitting indicated coordination of the azomethine nitrogen. Moreover, a low frequency shift (10-15 $cm^{-1}$) of the band due to the azomethine (HC=N) linkage at 1635 $cm^{-1}$ indicated involvement of the azomethine nitrogen in coordination. The appearance of weak, low frequency new bands at ~360 and ~455 $cm^{-1}$ were assigned[25] to metal-sulfur ν(M-S) in the thienyl and metal-oxygen ν(M-O) in the furanyl ligands. These bands were only observable in the spectra of the metal complexes and not in the spectra of their Schiff bases which in turn confirmed the participation of the heteroatoms X (S or O) in the coordination. These observations, in turn, suggested[22,26] a square planar geometry for the Cu(II) complexes (Fig. 3).

**NMR Spectra**

The NMR spectra of the free ligands and some of their metal complexes have been recorded in DMSO-$d_6$. The features of the free ligands are already reported[27] elsewhere which have shown the NMR spectra of the free ligands in support to the conclusions derived from the IR spectra as expected. In the spectra of the Cu(II) complexes (Table III), these proton signals appeared much more downfield, as expected, due to increased conjugation during coordination[28,29]

Table II. IR and UV-Visible Data of the Cu(II) Complexes

| No | IR ($cm^{-1}$) | $\lambda_{max}$ ($cm^{-1}$) |
|---|---|---|
| 1 | 3315 (b, OH), 1625 (s, HC=N), 1025 (m, N-N), 455 (m, M-O). | 14680, 16390, 27320 |
| 2 | 3320 (b, OH), 1620 (s, HC=N), 1025 (m, N-N), 455 (m, M-O). | 14675, 16385, 27335 |
| 3 | 3317 (b, OH), 1625 (s, HC=N), 1025 (m, N-N), 455 (m, M-O). | 14685, 16380, 27330 |
| 4 | 3315 (b, OH), 1625 (s, HC=N), 1025 (m, N-N), 455 (m, M-O). | 14680, 16395, 27335 |
| 5 | 3317 (b, OH), 1620 (s, HC=N), 1025 (m, N-N), 360 (m, M-S). | 14690, 16385, 27325 |
| 6 | 3317 (b, OH), 1620 (s, HC=N), 1025 (m, N-N), 360 (m, M-S). | 14685, 16380, 27335 |
| 7 | 3315 (b, OH), 1625 (s, HC=N), 1025 (m, N-N), 360 (m, M-S). | 14675, 16390, 27325 |
| 8 | 3320 (b, OH), 1625 (s, HC=N), 1025 (m, N-N), 360 (m, M-S). | 14680, 16385, 27330 |

s = sharp, m = medium, b = broad

Table III. NMR Spectral Data of the Cu(II) Complexes

| Complex | $^{1}H$ NMR (DMSO-$d_6$) (ppm) | $^{13}C$ NMR (DMSO-$d_6$) (ppm) |
|---|---|---|
| 1 | 4.8 (s, 1H, OH), 5.0-5.2 (m, 1H, furanyl), 5.4 (m, 1H, furanyl), 5.9 (m, 1H, furanyl), 8.2 (s, 1H, HC=N). | 82.8 (C-O), 105.9, 113.1, 121.8, 124.2 (furanyl),153.1 (HC=N). |
| 2 | 4.9 (s, 1H, OH), 5.1-5.3 (m, 1H, furanyl), 5.5 (m, 1H, furanyl), 5.9 (m, 1H, furanyl), 8.3 (s, 1H, HC=N). | 82.9 (C-O), 105.9, 113.3, 121.8, 124.3 (furanyl),153.2 (HC=N). |
| 3 | 4.8 (s, 1H, OH), 5.2-5.3 (m, 1H, furanyl), 5.4 (m, 1H, furanyl), 5.9 (m, 1H, furanyl), 8.2 (s, 1H, HC=N). | 82.8 (C-O), 105.9, 113.2, 121.9, 124.3 (furanyl),153.1 (HC=N). |
| 4 | 4.9 (s, 1H, OH), 5.1-5.2 (m, 1H, furanyl), 5.4 (m, 1H, furanyl), 5.9 (m, 1H, furanyl), 8.3 (s, 1H, HC=N). | 82.9 (C-O), 105.9, 113.2, 121.8, 124.2 (furanyl),153.3 (HC=N). |
| 5 | 4.7 (s, 1H, OH), 5.1-5.2 (m, 1H, thienyl), 5.3 (m, 1H, thienyl), 5.8 (m, 1H, thienyl), 8.1 (s, 1H, HC=N). | 82.6 (C-O), 105.8, 113.1, 121.7, 124.1 (thienyl),153.1 (HC=N). |
| 6 | 4.8 (s, 1H, OH), 5.1-5.2 (m, 1H, thienyl), 5.4 (m, 1H, thienyl), 5.8 (m, 1H, thienyl), 8.2 (s, 1H, HC=N). | 82.5 (C-O), 105.7, 113.3, 121.7, 124.1 (thienyl),153.2 (HC=N). |
| 7 | 4.8 (s, 1H, OH), 5.0-5.2 (m, 1H, thienyl), 5.4 (m, 1H, thienyl), 5.8 (m, 1H, thienyl), 8.3 (s, 1H, HC=N). | 82.8 (C-O), 105.8, 113.2, 121.7, 124.2 (thienyl),153.3 (HC=N). |
| 8 | 4.8 (s, 1H, OH), 5.1-5.3 (m, 1H, thienyl), 5.4 (m, 1H, thienyl), 5.7 (m, 1H, thienyl), 8.2 (s, 1H, HC=N). | 82.7 (C-O), 105.7, 113.3, 121.6, 124.1 (thienyl), 153.2 (HC=N). |

**Magnetic Moment and Electronic Spectra**

The UV-Visible spectral bands of the Cu(II) complexes are recorded in Table I. The copper(II) complexes exhibited[30] magnetic moment of 1.55-1.65 B.M at room temperature. These values are quite close to the spin-allowed values expected for a S=1/2 system and the complexes attain a square planar geometry around the copper(II) ion. These copper(II) complexes (Table II) display a broad band at ~14680 $cm^{-1}$ due to $^2B_{1g} \rightarrow {}^2E_g$ and two bands at ~16395 and ~27320 $cm^{-1}$ assigned[31,32] due to d-d transitions and a charge transfer band, confirming their square planar environment[33] (Fig. 3).

**Antibacterial Properties**

Antibacterial properties of the ligands and their metal complexes were studied against bacterial species *Escherichia coli, Pseudomonas aeruginosa, Staphylococcus aureus* and *Klebsiella pneumonae*. These were tested at a concentration of 30 µg/0.01 mL in DMF solution using a paper disc diffusion method devised and reported[34-37] earlier. The results of these studies reproduced in Table IV indicated that both the Schiff-base ligands and their metal complexes individually exhibited varying degrees of inhibitory effects on the growth of the testing bacterial species. The antibacterial results evidently show that the activity of the unchelated compounds became more pronounced and prominent when chelated with the metal ion. When the same metal chelate having different anions was individually screened, the degree of bectericidal activity/potency also varied.

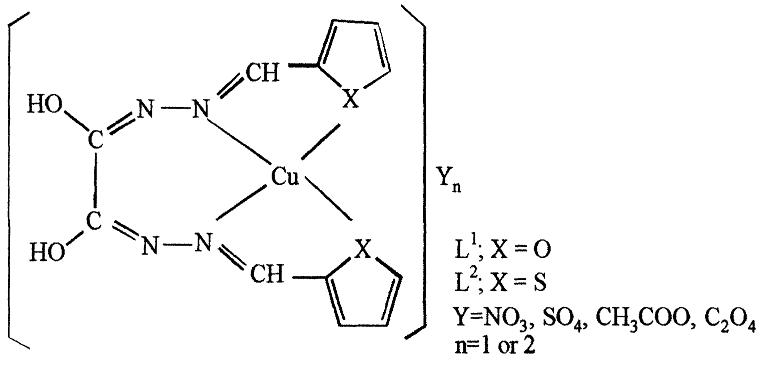

(Fig. 3) Proposed Structure of the Cu(II) Complex

From the obtained data, it was generally observed that the order of potency in comparison to the metal chelate having chloride anion evaluated and reported earlier[38] and to the results of the present studies against the same tested bacterial species under the same conditions were found to follow the order as

$$NO_3 > C_2O_4 > CH_3CO_2 > Cl > SO_4$$

On the basis of these results, it is therefore, strongly claimed[39,40] that different anions dominantly effect the biological behavior of the metal chelates. It is, however, expected that factors such as solubility, conductivity, dipole moment and cell permeability mechanisms are certainly influenced by the presence of these anions in the chelate and may cause in increasing this activity. These studies provide a useful information about the biological activity of compounds influenced by the anions which stay outside the coordination sphere of the chelated complex.

**ACKNOWLEDGEMENT**

The author gratefully acknowledges the Department of Microbiology, Qaid-e-Azam Medical College, Bahawalpur, Pakistan, for its help in undertaking the antibacterial studies.

Table IV Antibacterial Activity Data

| Ligand/ complex | Microbial Species a | b | c | d |
|---|---|---|---|---|
| $L^1$ | ++ | ++ | + | ++ |
| $L^2$ | + | ++ | - | ++ |
| 1 | ++++ | +++ | ++ | ++ |
| 2 | +++ | +++ | +++ | ++ |
| 3 | +++ | +++ | ++ | +++ |
| 4 | +++ | ++ | +++ | ++ |
| 5 | +++ | ++ | +++ | +++ |
| 6 | ++ | +++ | + | ++ |
| 7 | +++ | +++ | ++ | +++ |
| 8 | ++ | +++ | ++ | +++ |

a=*Escherichia coli*, b= *Staphylococcus aureus*, c= *Pseudomonas aeruginosa* d=*Klebsiella pneumonae*. Inhibition zone diameter mm (% inhibition): +, 6-10 (27-45 %); ++, 10-14 (45-64 %); +++, 14-18 (64-82 %); ++++, 18-22 (82-100 %). Percent inhibition values are relative to inhibition zone (22 mm) of the most active compound with 100 % inhibition.

### REFERENCES

1. T. B. Murphy, D. K. Johnson, N. J. Rose, A. Aruffo and V. Schomaker, *Inorg. Chim. Acta*, **70**, 151 (1983).
2. E. B. Flescher, D. Jeter and R. Flortan, *Inorg. Chem.*, **13**, 1042 (1972).
3. E. B. Flescher and M. B. Lawson, *Inorg. Chem.*, **11**, 2772 (1972).
4. C. L. Clein, E. B. Seven, C. J. O'Conner, R. J. Majestes and L. M. Trefonas, *Inorg. Chim. Acta*, **71**, 130 (1984).
5. C. Pelizzi, G. Pelizzi and F. Vitali, *J. Chem. Soc Dalton. Trans.*, 177, 1987.
6. C. Pelizzi, G. Pelizzi, G. Predieri and S. Resolva, *J. Chem. Soc Dalton Trans.*, 1349, (1982).
7. M. B. Hursthouse, S. Anarasiri, A. Jayweera and A. Quick, *J. Chem. Soc Dalton Trans.*, 279 (1979).
8. R. Haran, J. Gairin and G. Commenges, *Inorg. Chim. Acta*, **46**, 63 (1980).
9. J. R. Dimmock, G. B. Baker and W. G. Taylor, *Can. J. Pharm. Sci.*, **7**, 100 (1972).
10. K. K. Narang and M. Singh, *Synth. React. Inorg. Met-Org. Chem.*, **15**, 821 (1985).
11. R. C. Aggarwal and K. K. Narang, *Ind. J. Chem.*, **19A**, 64 (1976).
12. R. Gopal, V. N. Mishra and K. K. Narang, *Ind. J. Chem.*, **14A**, 364 (1976).
13. D. K. Johnson, T. B. Murphy, N. J. Rose, W. H. Goodwin and L. Pickart, *Inorg. Chim. Acta.*, **67**, 159 (1982).
14. L. Pickart, W. H. Goodwin, W. Burgua, T. B. Murphy and D. K. Johnson, *Biochem. Pharmacol.*, **32**, 3868 (1983).
15. Z. H. Chohan and C. T. Supuran, *Main Group Met. Chem.*, **24**, 399 (2001).
16. Z. H. Chohan, *Synth. React. Inorg. Met-Org. Chem*, **31**, *1 (2001)*.
17. Z. H. Chohan, M. A. Farooq and M. S. Iqbal, *Met. Based Drugs*, **7**, 133 (2000).
18. Z. H. Chohan and S. K. A. Sherazi, *Synth. React. Inorg. Met-Org. Chem*, **29**, *105 (1999)*.
19. W. J. Geary, *Chem. Rev.*, **7**, 81 (1971).
20. K. Burger, I. Ruff and F. Ruff, *J. Inorg. Nucl. Chem.*, **27**, 179 (1965).
21. C. Pelizzi and G. Pelizzi, *Inorg. Chim. Acta*, **18**, 39 (1976).
22. R. C. Aggarwal, N. K. Singh and R. P. Singh, *Inorg. Chem.*, **70**, 2794 (1981).
23. L. J. Bellamy, "*The Infrared Spectra of Complex Molecules*", 3rd Ed, Methuen, London, (1966).
24. S. R. Patil, U. N. Kantak and D. N. Sen, *Inorg. Chim. Acta*, **63**, 261 (1982).
25. K. Dey and D. Bandyopadhyay, *Trans. Met. Chem.*, **16**, 267 (1991).
26. K. Nakamoto, "*Infrared Spectra of Inorganic and Coordination Compounds*", 2nd Ed, Wiley Interscience, New York, (1970).
27. Z. H. Chohan and M. A. Farooq, *Synth. React. Inorg. Met- Org. Chem, (In press)*.
28. D. J. Pasto and C. R. Johnson, "*Organic Structure Determination*", Prentice-Hall International, London, (1969).
29. D. L. Pavia, G. M. Lampman and G. S. Kriz, "*Introduction to Spectroscopy*", Harcourt Brace and Comp, Florida, U.S.A, (1996).
30. A. B. P. Lever, J. Lewis and R. S. Nyholm, *J. Chem. Soc.*, 2552, 1963.
31. A. B. P. Lever, "*Inorganic Electronic Spectroscopy*", Elsevier, Amsterdam, (1984).
32. D. W. Meek, R. S. Drago and T. S. Piper, *Inorg. Chem.*, **1**, 285 (1962).

33. C. J. Balhausen, "*An Introduction to Ligand Field*", McGraw Hill, New York, (1962).
34. Z. H. Chohan and A. Rauf, *J. Inorg. Biochem*, **46**: 41 (1992).
35. Z. H. Chohan, *Met.-Based Drugs*, 6: 75 (1999).
36. Z. H. Chohan, M. F. Jaffery and C. T. Supuran, *Met. Based Drugs*, **8**, 95 (2001).
37. Z. H. Chohan and M. A. Farooq, *Synth. React. Inorg. Met- Org. Chem*, (In Press).
38. Z. H. Chohan and M. Praveen, *J. Chem. Soc. Pak*, **22**, 186 (2000).
39. Z. H. Chohan and S. K. A. Sherazi, *Met. Based Drugs*, **4**, 327 (1997).
40. Z. H. Chohan and M. Praveen, *Met. Based Drugs*, **6**, 95 (1999).

**Received: July 22, 2000 – Accepted: August 31, 2000 – Accepted in publishable format: September 13, 2001**

